# Sterile keratolysis following pars plana vitrectomy for retinal detachment

**DOI:** 10.1007/s10792-025-03532-3

**Published:** 2025-05-08

**Authors:** Anna Hillenmayer, Christian M. Wertheimer, Efstathios Vounotrypidis, Armin Wolf, Melih Parlak

**Affiliations:** Department of Ophthalmology, University Hospital Ulm, Prittwitzstr. 43, 89075 Ulm, Germany

**Keywords:** Retinal detachment, Corneal ulcer, Keratolysis, Silicone tamponade, Vitrectomy

## Abstract

**Purpose:**

Pars plana vitrectomy (PPV) with various forms of tamponade and retinopexy is often the primary treatment for retinal detachment. However, a rare but serious complication is sterile keratolysis. We therefore aimed to evaluate the prevalence and potential risk factors for the development of corneal ulceration following vitreoretinal surgery for retinal detachment.

**Methods:**

This is a single-centre retrospective study including 14 cases of patients presenting to our department with sterile keratolysis involving the stroma after one or more PPVs for retinal detachment or vitreous hemorrhage. Time of primary procedure, time of onset of corneal complications, comorbidities, type of tamponade used, use of endophotocoagulation, cryoretinopexy and number of surgeries were recorded. Patients with additional comorbidities confounding a possible correlation were excluded from the case series.

**Results:**

A total of 14 patients were identified with corneal complications after pars plana vitrectomy for retinal detachment. Multiple vitrectomies were performed in 86% (12/14) of the cases. Surgical treatment consisted of six (43%) perforating keratoplasties and seven (50%) amniotic membrane keratoplasties in all but one patient. At an average of three months after the onset of corneal symptoms, the first corneal surgery was performed. Repeated corneal surgery was required in 4 patients (29%) and consisted of two penetrating keratoplasties and four amniotic membrane transplantation. Visual acuity at the first presentation of corneal complications was reduced (2.1 ± 0.6 logMAR), but was not statistically different from the visual acuity at baseline (1.6 ± 0.7 logMAR). At the last follow-up, visual acuity remained reduced at 1.8 ± 0.8 logMAR (*p* = 0.2).

**Conclusions:**

The risk of sterile keratolysis seems to increase with excessive laser or cryo-retinopexy, use of silicone oil and repeated procedures. The initial vitrectomy was a complex surgery in all cases and required a longer operating time. Ciliary nerve damage of neurotrophic origin may be the cause of sterile keratolysis, and controlled retinopexy sparing the long ciliary nerves and avoiding cryotherapy may reduce the risk. Controlled studies are needed to investigate the causality between vitreoretinal surgery and sterile keratolysis.

## Introduction

Pars plana vitrectomy (PPV) is a common treatment option for various retinal diseases, particularly retinal detachments, which are currently experiencing an increase in incidence. [1, 2] The procedure can result in complications in the cornea, either through direct trauma such as intraoperative abrasio corneae, toxicity, or disorders in structures that supply the cornea, such as nerves, aqueous humor, and vessels. [3, 3].

Corneal complications such as corneal epithelial defects, corneal edema, or superficial punctate keratopathy in the early postoperative period within a few weeks after PPV are commonly observed [4]. These complications are typically managed medically and resolve without long-term consequences. Severe corneal complications that involve the corneal stroma are a rare occurrence, and there is a paucity of evidence of a few described cases regarding the entity [3–5].

Sterile keratolysis is a rare but serious complication of pars plana vitrectomy (PPV) for retinal detachment. It can result in persistent vision loss. Consequently, we identified fourteen patients who suffered from this complication and the following is to describe the clinical course and to identify potential risk factors for its development.

## Methods

### Study design

Fourteen patients who underwent PPV between 2010 and 2023 for rhegmatogenous and tractional retinal detachment at our tertiary university hospital were identified as having corneal complications and were included in this retrospective cohort study. This study was approved by the Ethics Committee of the University of Ulm (ID 441/23) and adhered to the tenets of the Declaration of Helsinki.

### Inclusion and exclusion criteria

The aim was to include only patients with a high probability that the sterile keratolysis was causally related to PPV for retinal detachment and that the corneal stroma was involved. To avoid confounding, this resulted in the following exclusion criterion: any predisposing ocular surface disease that commonly leads to corneal ulceration was excluded, as was any infectious keratitis. Patients who had undergone corneal surgery prior to PPV, for example for pterygium or prior glaucoma surgeries predisposing for ocular surface disease, were also excluded.

### Surgical procedure

All patients underwent one or more standard 23 or 25 gauge pars plana vitrectomy (PPV) procedures, with or without phacoemulsification, under appropriate anesthesia. The surgeons proceeded to implant a range of intraocular lenses into the capsular bag. In cases of epiretinal gliotic, internal limiting membrane or vitreoretinal membrane peeling was performed with the use of brilliant blue dye and end-grasping forceps, if necessary. Endolaser photocoagulation or cryoretinopexy was employed when necessary and in accordance with the surgeon’s discretion. A range of endotamponades were utilized, including balanced salt solution, silicone oil, gas (SF6, C2F6, C3F8), and air, contingent on the specific pathology, other individual factors, and the surgeon’s preference. All patients received topical antibiotics and topical steroids postoperatively.

### Statistical analysis

All calculations and data collection were performed in Excel 365 (Microsoft, Redmond, WA, USA). Graphs were generated using GraphPad PRISM 9 (GraphPad Software, Inc., San Diego, CA, USA). ANOVA with the least significant difference post hoc test was performed with GraphPad PRISM 9 to compare between groups due to the small sample size. A *p* value < 0.05 was considered statistically significant.

## Results

### Demographics

In our retrospective chart review, we were able to identify 14 patients who suffered from severe corneal stromal complications leading to inpatient admission. The majority of eleven patients (79%) were biologically male and the mean age at first presentation was 67 ± 14 years. The diagnosis leading to initial retinal surgery was retinal detachment. Twelve patients (86%) underwent pars plana vitrectomy, one patient (7%) underwent retinal cryoretinopexy and one patient (7%) underwent scleral buckling. Mean visual acuity at presentation was significantly reduced to 1.6 ± 0.7 logMAR, with one patient (7%) able to count fingers, five (36%) able to recognize hand movements, and one (7%) able to perceive light only.

### Follow-up

**Fig. 1:**
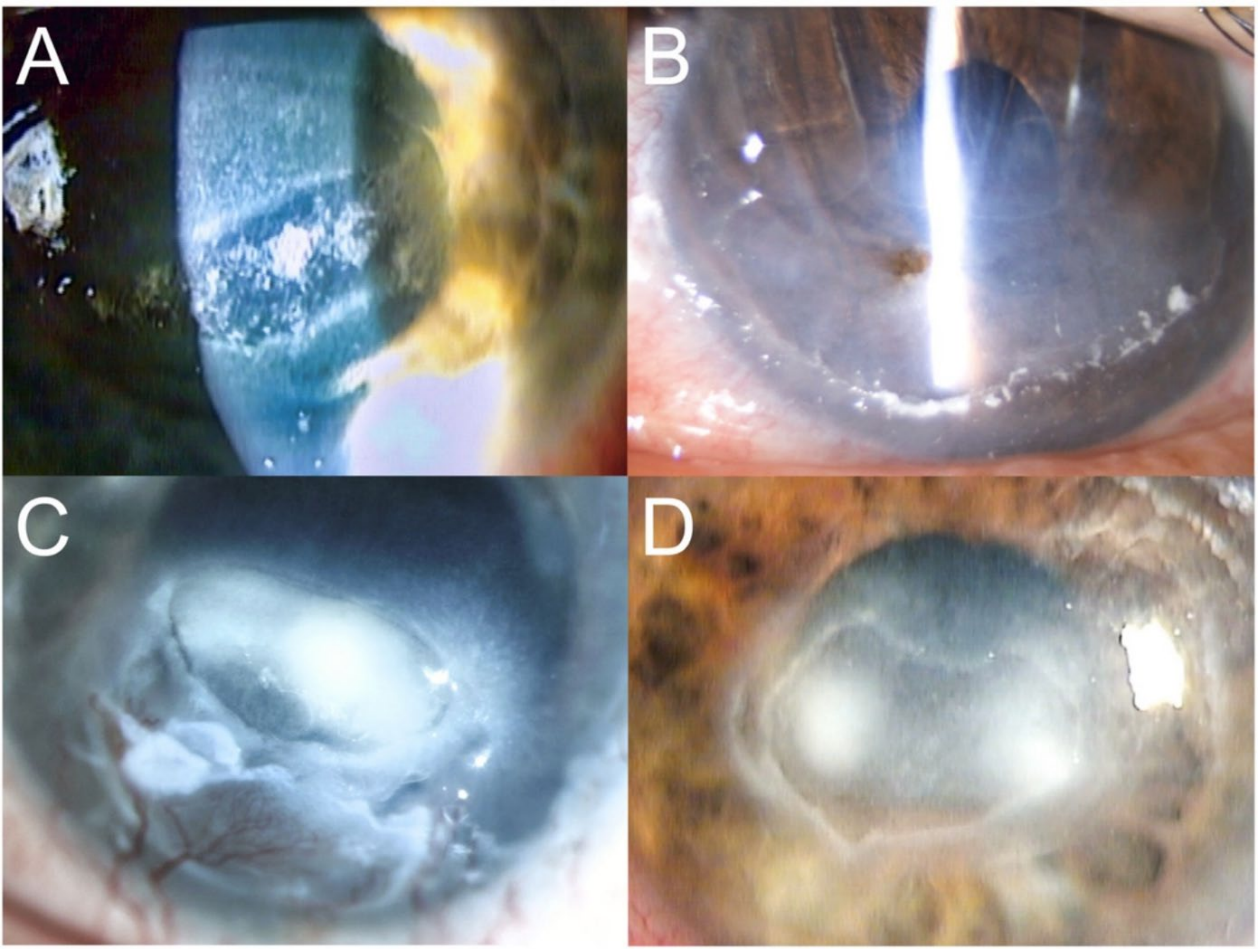
Exemplary anterior segment photographs of 4 patients with corneal complications following pars plana vitrectomy. Three patients (including C) had corneal perforations that necessitated penetrating keratoplasty. The remaining eleven patients (including A, B and D presented with corneal stromal ulceration, which required either penetrating keratoplasty or amniotic membrane transplantation

Mean follow-up was 60 ± 56 months and patients presented with corneal complications relatively late after the initial retinal surgery, at approximately 41 ± 54 months. All but two eyes received more than one pars Plana vitrectomy, for a total of 3 ± 1.5 for all patients. Three (21%) had corneal perforation and all others had corneal stromal ulceration (Fig. 1) without cofounding factors being detected for ocular surface disease.

**Fig. 2:**
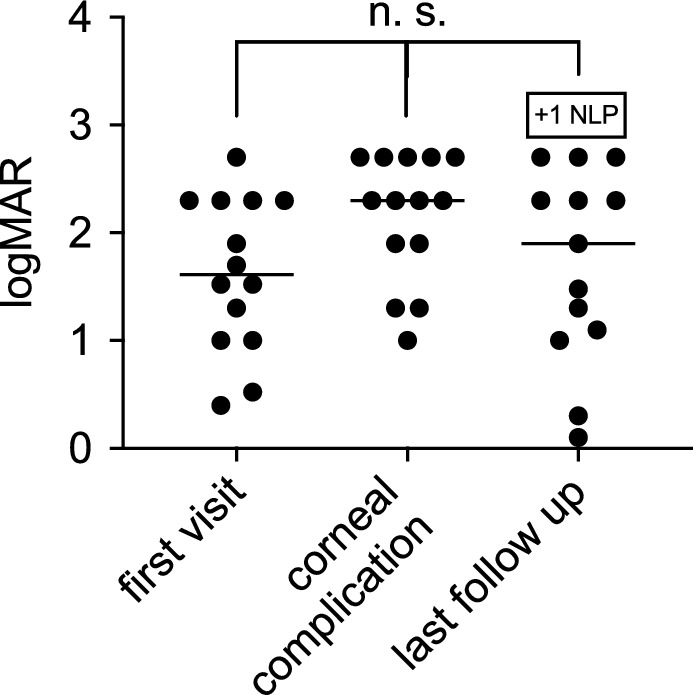
Development of visual acuity. The patient’s visual acuity was found to be significantly reduced at the initial presentation, with a value of 1.6 ± 0.7 logMAR, due to retinal pathology. However, visual acuity at the initial presentation of corneal complications was reduced (2.1 ± 0.6 logMAR), but not statistically different from the visual acuity at baseline and remained reduced at the final follow-up at 1.8 ± 0.8 logMAR (*p* = 0.1)

All but one patient underwent surgical treatment, consisting of six (43%) penetrating keratoplasties and seven (50%) amniotic membrane transplantations. The first corneal surgery was performed a mean of three months after the initial presentation of corneal symptoms following prior retinal surgery. Repeated corneal surgery was required in four patients (29%) and consisted of two penetrating keratoplasties and four amniotic membrane transplantations (29%) (shown in Fig. 1). Visual acuity at the first presentation of corneal complications was reduced (2.1 ± 0.6 logMAR), but not statistically different from baseline, and remained reduced at the last follow-up at 1.8 ± 0.8 logMAR (*p* = 0.1) (shown in Fig. 2).

### Potential precipitating factors

In addition to multiple pars plana vitrectomies and retinal detachment, other factors may have contributed to the development of corneal disease. All 14 eyes underwent endolaser treatment and half of the eyes had documented 360° circular laser retinopexy. Nine (64%) eyes underwent cryo-retinopexy for retinal holes. Silicone oil tamponade was recorded in 11 (78%) eyes, with the oil remaining in the eye for a prolonged period of 15 ± 31 months before the onset of corneal symptoms. Six (43%) of these eyes had documented migration of the oil into the anterior chamber. In addition, four (29%) eyes had glaucoma and required chronic medication. One patient (7%) had diabetes without microvascular complications. (Table 1).

**Table 1:** Key characteristics of all 14 cases

Demographics		
Number of patients	14	11 male
		3 female
Age at presentation (years)	67 ± 14	
Surgery for retinal detachment	12 pars plana vitrectomies 1 retinal cryopexy 1 scleral buckling	3 ± 1.5 mean retinal surgeries
Mean follow-up time (months)	60 ± 56	
Corneal complications		
Time to corneal complication	∼41 ± 54 months post-retinal surgery	3 corneal perforations 11 corneal stromal ulcerations
Corneal surgical treatment	6 perforating keratoplasties 7 amniotic membrane transplantations	2 repeated perforating keratoplasties 2 repeated amniotic membrane transplantations
Time of first corneal surgery	3 months after onset of corneal symptoms	
Mean visual acuity (logMAR)		
At presentation	1.6 ± 0.7	1 = 2.3
First corneal complication	2.1 ± 0.6	1 = 2.7
Last follow up	1.8 ± 0.8	5 = 1.9
Case characteristics		
Endolasar treatment	14	7 with 360° circular retinopaxy
Cryo-retinopexy	9	
Silicone oil tamponade	11	15 ± 31 months of tamponade before onset of corneal symptoms
Glaucoma with topical treatment	4	6 × migration into anterior chamber
Diabetic retinopath	1	

86% of included pateints underwent multiple vitrectomies. Corneal complications were addressed through perforating keratoplasty (43%) and amniotic membrane keratoplasty (50%), with the initial surgery taking place on average three months after the onset of symptoms. Four patients required repeated corneal surgeries. Initial visual acuity was 1.6 ± 0.7 logMAR, worsening to 2.1 ± 0.6 logMAR with corneal complications, though this decline was not statistically significant. The final visual acuity showed marginal improvement, reaching 1.8 ± 0.8 logMAR. The treatment modalities employed included endolaser treatment (100%), circular laser retinopexy (50%), cryo-retinopexy (64%), and silicone oil tamponade (78%), with oil persisting for 15 ± 31 months in some cases and migrating to the anterior chamber in 43%

### Single case example

**Fig. 3:**
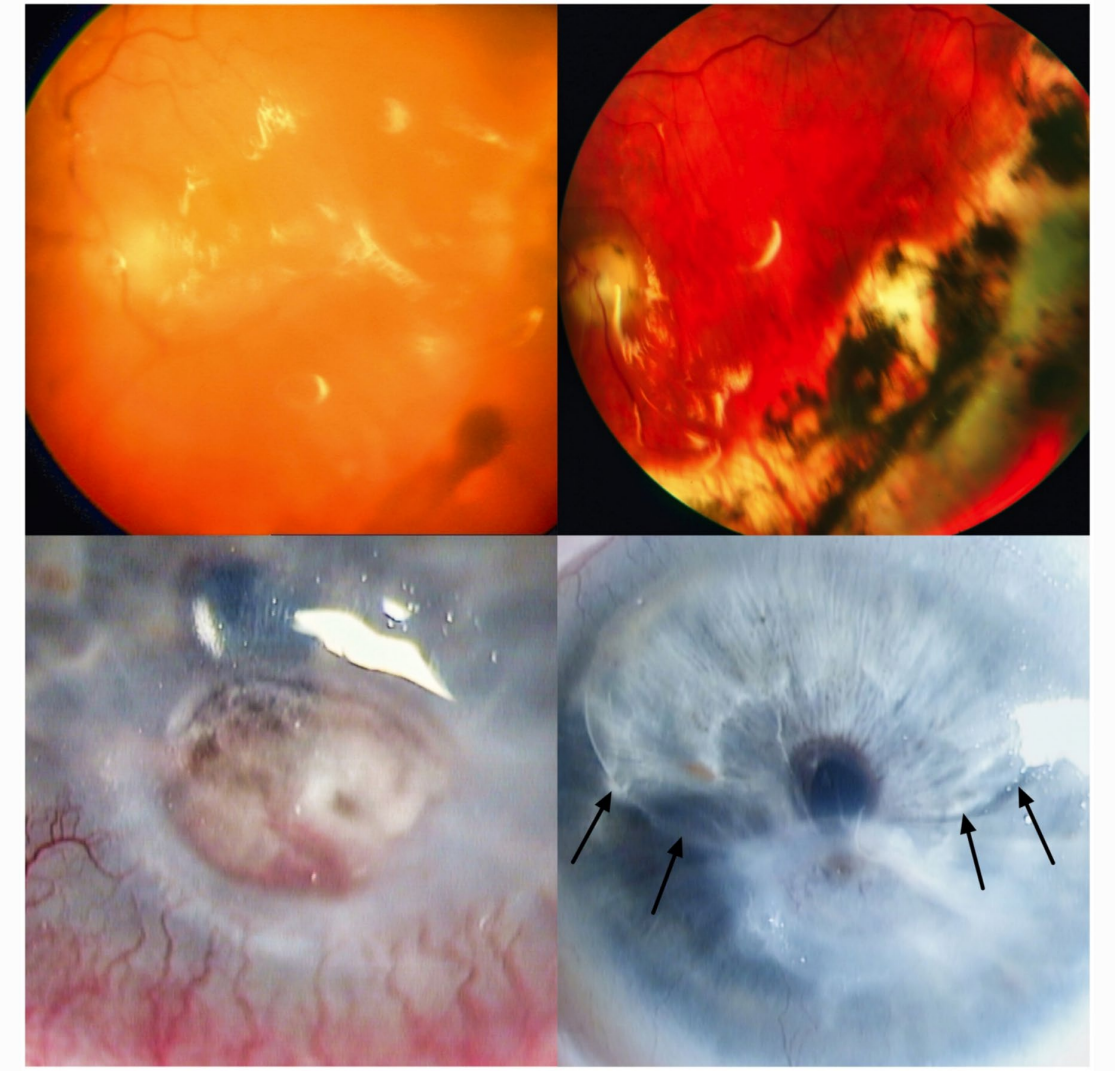
Exemplary case: Fundus photographs after the first pars plana vitrectomy with silicone oil (left photograph) for tractional retinal detachment with PVR C and five years later (right photograph). Ten years after the primary surgery, the patient presented with a corneal ulcer with iris incarceration of probable neurotrophic origin (left), which was treated with amniotic membrane inlay and overlay patch. At the patient’s request, no further surgery was performed for the remaining anterior synechiae and intracameral silicone oil (arrows, right photograph)

A 54-year-old man presented to our outpatient clinic with a seven-week history of decreased vision in the left eye and no known ocular history. Fundus examination revealed a tractional circumferential retinal detachment with proliferative vitreoretinopathy grade C. Visual acuity was light perception in the affected left eye and 0.1 logMAR in the right eye, intraocular pressure was 12 and 15 mmHg, respectively. Primary treatment was combined phacovitrectomy with 360° circular retinectomy, exocryocoagulation, silicone oil tamponade and encircling band. Two further surgeries were performed for iris capture, posterior synechiae and secondary capsule opacification. Eight months later, he underwent another vitrectomy with membrane peeling, retinotomy, endolaser and silicone oil exchange due to retinal detachment and excessive retinal membrane traction. Over the next few years, the right eye underwent also multiple retinal detachment surgeries, while the left eye remained stable with reduced visual prognosis and residual intravitreal silicone oil. Ten years after the primary surgery, the patient presented as an emergency case with a corneal ulcer with iris incarceration of probable neurotrophic origin. Vision was reduced to no light perception. The primary treatment was an amniotic membrane patch (inlay and overlay) which achieved complete epithelial closure. At the patient’s request, no further surgery was performed for the remaining anterior synechiae and intracameral silicone oil, and phthisis bulbi has since developed (shown in Fig. 3).

## Discussion

Corneal complications following pars plana vitrectomy involving the corneal stroma appear to be uncommon. Our analysis identified fourteen patients who experienced such complications. The majority of these patients also had a complex course of retinal disease with multiple and extensive surgery, and all had retinal detachment as the indication for the initial pars plana vitrectomy. Outpatient medical treatment was insufficient to treat the corneal complications, and surgery with keratoplasty or amniotic membrane was necessary in all but one patient.

The exact mechanism of corneal ulceration could not be determined from the available data. It can be speculated that neurotrophic keratopathy may be a reason for a reduction or absence of corneal sensitivity leading to epithelial defect, stromal ulceration and eventually corneal perforation [6]. In another study, corneal nerve fibre length was shown to be reduced after pars plana vitrectomy, especially in patients with early postoperative corneal complications [7]. Corneal ulceration due to neurotrophic keratitis has been reported in five patients without diabetes five to ten weeks after vitrectomy [8]. Another report of three patients is consistent with this hypothesis, though corneal complications presented significantly later [3].

Several mechanisms of damage during pars plana vitrectomy may be a possible reason for the occurrence of neurotrophic keratopathy. Damage to the long ciliary nerve during extensive laser coagulation has been observed as a risk factor in patients thought to have developed neurotrophic keratopathy in the early postoperative period [5]. Notably, these patients did not have diabetes. Loss of corneal sensation was also reported in another study of argon laser coagulation in diabetes [8]. A large number of our patients underwent extensive laser coagulation and cryocoagulation.

Silicone oil tamponade has also been associated with corneal complications after pars plana vitrectomy [9]. In our study, three-quarters of the patients had silicone oil tamponade, often for a prolonged period and with anterior chamber migration. In another study of five patients who developed corneal ulceration after vitrectomy, all patients had silicone oil tamponade [5]. Silicone oil is supposed to be non-toxic, but it is associated with several ocular complications. These complications include optic nerve damage, glaucoma and retinal infiltration [10]. Silicone oil also causes corneal sequelae known as silicone oil keratopathy, which presents with band keratopathy, corneal thinning, retrocorneal membrane formation and endothelial cell loss [11].

Another possible cause is toxicity due to perioperative administration of topical medications. A report of five ulcers [12] and another of three ulcers [3] have linked the use of non-steroidal anti-inflammatory drugs to the development of ulcers. In our study, four eyes exhibited glaucoma and required chronic medication, and all patients received anti-inflammatory drugs after pars plana vitrectomy. Interestingly, in average, the corneal complications manifested relatively late after the initial retinal procedure, at approximately 41 ± 54 months.

The present case series demonstrates the multifactorial nature of the development of sterile keratolysis after pars plana vitrectomy. Early identification of risk factors may prevent rapid and severe progression to corneal ulceration. In addition to general protective measures such as corneal lubrication, postoperative ocular surface protection and intraocular pressure management, prophylactic measures may include careful risk–benefit assessment of the use of excessive retinal laser coagulation, optimisation of the choice and duration of tamponade and early recognition of corneal decompensation. Early intervention with bandage contact lenses, amniotic membrane transplantation or targeted therapies for neurotrophic keratopathy may also be considered. Patients at risk should probably be assessed for individual risk with individualised therapeutic strategies to prevent severe keratolysis.

Limitations of the study include the relatively small number of cases and the unknown exact number of total pars plana vitrectomies performed during this time. Although we expect the incidence of corneal ulceration after vitrectomy to be very low, an exact rate cannot be determined. With an approximate of 5000 patients having undergone PPV for retinal detachment at our center from 2010 to 2023, a possible 0,2% would present with sterile keratolysis. The very small sample size, mainly due to the rarity of ulceration after pars plana vitrectomy, may lead to underpowering in statistical testing and future studies, possibly multicentric, will need to determine differences in larger sample sizes. Additionally, potential confounders may have been overlooked due to the retrospective nature of the study. Furthermore, given the potential significance of developing neurotrophic keratopathy as the underlying cause, the assessment of corneal sensitivity may have been a valuable approach in identifying and formulating early intervention strategies. However, due to the retrospective nature of the study, this data is not available but should be included in future studies.

## Conclusion

Retinal detachments with frequently necessitate multiple surgical procedures, including extensive laser photocoagulation, cryotherapy, large retinectomies, and silicone oil tamponade. In the course of our study, we identified 14 patients who had experienced severe corneal complications, including sterile keratolysis, as a result of complex retinal surgery. While the incidence of these complications is relatively low, it is nevertheless important to ensure that patients are fully informed about this rare but potentially serious adverse event. Regular follow-up is essential to address any neurotrophic corneal issues that may arise early on and to preserve the visual acuity of patients who have already experienced a significant loss of vision. Further research, particularly multicentric studies, is warranted to gain a deeper understanding of these complications and to develop more effective strategies for their management.

## Data Availability

No datasets were generated or analysed during the current study.

## References

[1] Nielsen BR, Alberti M, Bjerrum SS, la Cour M (2020) The incidence of rhegmatogenous retinal detachment is increasing. Acta Ophthalmol 98(6):603–606. 10.1111/aos.14380 32086859 10.1111/aos.14380

[2] Alfaar AS, Wiedemann P, Rehak M, Wolf A (2024) The rising tide of rhegmatogenous retinal detachment in Germany: a nationwide analysis of the incidence, from 2005 to 2021. Graefe’s Arch Clin Exp Ophthalmol 262(8):2431–2438. 10.1007/s00417-024-06392-2 38466396 10.1007/s00417-024-06392-2 PMC11271417

[3] Kurt RA, Sonmez B, Kapran Z (2021) Neurotrophic keratopathy after retinal detachment surgery combined with Endolaser photocoagulation. Retin Cases Brief Rep 15(4):479–481. 10.1097/ICB.0000000000000832 30300314 10.1097/ICB.0000000000000832

[4] Chen HF, Yeung L, Yang KJ, Sun CC (2016) Persistent corneal epithelial defect after pars Plana vitrectomy. Retina 36(1):148–155. 10.1097/IAE.0000000000000657 26166798 10.1097/IAE.0000000000000657

[5] Banerjee PJ, Chandra A, Sullivan PM, Charteris DG (2014) Neurotrophic corneal ulceration after retinal detachment surgery with retinectomy and endolaser: a case series. JAMA Ophthalmol 132(6):750–752. 10.1001/jamaophthalmol.2014.280 24743924 10.1001/jamaophthalmol.2014.280

[6] NaPier E, Camacho M, McDevitt TF, Sweeney AR (2022) Neurotrophic keratopathy: current challenges and future prospects. Ann Med 54(1):666–673. 10.1080/07853890.2022.2045035 35243932 10.1080/07853890.2022.2045035 PMC8903790

[7] Lin T, Ye H, Pazo EE, Dai G, Xia Y, He W (2021) Corneal nerves alteration associated with corneal complications after pars Plana vitrectomy. Korean J Ophthalmol 35(4):255–260. 10.3341/kjo.2021.0048 34162192 10.3341/kjo.2021.0048 PMC8357606

[8] Menchini U, Scialdone A, Pietroni C, Carones F, Brancato R (1990) Argon versus krypton panretinal photocoagulation side effects on the anterior segment. Ophthalmologica 201(2):66–70. 10.1159/000310129 2234817 10.1159/000310129

[9] Green K, Cheeks L, Stewart DA, Trask D (1992) Role of toxic ingredients in silicone oils in the induction of increased corneal endothelial permeability. Lens Eye Toxic Res 9:377–384 1284519

[10] Yang CS, Chen KH, Hsu WM, Li YS (2008) Cytotoxicity of silicone oil on cultivated human corneal endothelium. Eye (Lond) 22(2):282–288. 10.1038/sj.eye.6702962 17721498 10.1038/sj.eye.6702962

[11] Foulks GN, Hatchell DL, Proia AD, Klintworth GK (1991) Histopathology of silicone oil keratopathy in humans. Cornea 10(1):29–37. 10.1097/00003226-199110010-00007 2019104

[12] Fossati G, Bartoli E, Montericcio A, Buzzi M, Barone G, Santoru F, Allegrini D, Romano MR, Panico C (2024) Neurotrophic keratopathy after wide retinal endolaser and postoperative ketorolac eye drops: A case series. Eur J Ophthalmol. 10.1177/11206721241228005 38254249 10.1177/11206721241228005

